# Liquid Biopsy-Guided Surgical Decision-Making in Oligometastatic Non-Small Cell Lung Cancer: Toward Dynamic Risk Stratification in the Multidisciplinary Era

**DOI:** 10.3390/cancers18203338

**Published:** 2026-10-16

**Authors:** Dimitrios E. Magouliotis, Vasiliki Androutsopoulou, Prokopis-Andreas Zotos, Namariq Abbaker, Ugo Cioffi, Fabrizio Minervini, Marco Scarci

**Affiliations:** 1 Department of Cardiac Surgery Research, Lankenau Institute for Medical Research, Main Line Health, Wynnewood, PA 19096, USA; magouliotisd@mlhs.org; 2 Department of Cardiothoracic Surgery, University of Thessaly, Biopolis, 41110 Larissa, Greece; androutsopoulouvasiliki@uth.gr (V.A.); zotospro@hotmail.com (P.-A.Z.); 3 Department of Surgery and Cancer, Hammersmith Hospital, Imperial College Healthcare NHS Trust, London W12 0HS, UK; n.abbaker@nhs.net; 4 Department of Surgery, University of Milan, 20157 Milan, Italy; ugo.cioffi@guest.unimi.it; 5 Department of Thoracic Surgery, Cantonal Hospital Lucerne, 6000 Lucerne, Switzerland; fabrizio.minervini@luks.ch

**Keywords:** oligometastatic disease, non-small cell lung cancer, circulating tumor DNA, liquid biopsy, minimal residual disease, thoracic surgery, multidisciplinary team, dynamic risk stratification

## Abstract

Oligometastatic non-small cell lung cancer (NSCLC) is defined anatomically by a threshold of five or fewer metastatic lesions across three or fewer organs. That definition is a proxy for a biological question it cannot answer: whether the metastatic process has been arrested at a stage where eradication of all detectable disease is curative. Randomized phase II trials of local consolidative therapy have shown median progression-free survival gains of 6.2 and 8.0 months and, in one trial, a median overall survival of 41.2 months vs. 17.0 months, yet median progression-free survival with local therapy did not exceed 14.2 months, and the phase II/III NRG-LU002 trial found no significant survival benefit from adding local therapy to maintenance therapy. Circulating tumor DNA (ctDNA) measures molecular disease burden. In resectable NSCLC, preoperative ctDNA detection is associated with a higher risk of relapse; in oligometastatic disease, the baseline sample is instead the quantitative reference for response. ctDNA kinetics during induction therapy track systemic response before any operation, although no response threshold has been validated, and postoperative molecular residual disease anticipates radiological relapse by a median of approximately 5 months. This review synthesizes the evidence linking liquid biopsy to surgical decision-making and proposes, as a hypothesis for prospective testing, a dynamic risk stratification framework anchored on five plasma sampling points. We separate established prognostic evidence from untested hypotheses, address oncogene-driven and other oligometastatic states, and define the assay requirements, confounders and trial designs needed before ctDNA can select patients for or against thoracic resection.

## Simple Summary

A minority of patients whose lung cancer has spread to a small number of distant sites can be cured when every visible deposit is removed or irradiated. Identifying those patients remains the central problem. Current practice counts lesions on scans, and counting cannot separate a tumor that has genuinely seeded two sites from one that has already seeded many sites too small to see. Circulating tumor DNA is released into the bloodstream by cancer cells, is measured from a routine blood draw, and tracks the quantity of disease in the body as treatment proceeds. This review examines how repeated blood sampling before, during and after treatment measures disease burden and the risk of relapse, and whether that information could identify the patients who benefit most from an operation. We propose a framework and set out the clinical trials needed to test it before it changes practice.

## 1. Introduction

Hellman and Weichselbaum proposed in 1995 that metastatic spread is a spectrum rather than a binary state, and that an intermediate condition exists in which the number and location of metastases are limited and the disease remains amenable to cure by local treatment [1]. Three decades later, oligometastatic NSCLC is a recognized clinical entity with a formal definition: five or fewer metastatic lesions distributed across three or fewer organs. Mediastinal nodal involvement is not counted as a metastatic site, staging with fluorodeoxyglucose positron emission tomography with computed tomography (FDG PET-CT) and brain imaging is mandatory, and at least one metastatic deposit must be confirmed histologically unless the risks outweigh the benefit [2]. The European Society for Radiotherapy and Oncology and the European Organisation for Research and Treatment of Cancer subsequently produced a nomenclature that separates genuine from induced oligometastatic disease and subclassifies it as oligorecurrence, oligoprogression or oligopersistence [3].

The clinical case for radical local treatment rests on three randomized phase II trials. Gomez and colleagues randomized patients with three or fewer metastases and no progression after first-line systemic therapy to local consolidative therapy or to maintenance therapy alone and reported median progression-free survival of 11.9 months vs. 3.9 months [4]. With a median follow-up of 38.8 months, the same trial showed median overall survival of 41.2 months in the local consolidative therapy arm vs. 17.0 months in the control arm [5]. Iyengar and colleagues randomized patients with up to five metastatic sites to stereotactic ablative radiotherapy followed by maintenance chemotherapy or to maintenance chemotherapy alone and reported median progression-free survival of 9.7 months vs. 3.5 months [6]. SABR-COMET, which enrolled patients with one to five metastases across mixed primary histologies, reported median overall survival of 41 months vs. 28 months and, at extended follow-up, five-year overall survival of 42.3% vs. 17.7% [7,8].

These results establish that local therapy benefits a selected population. They do not establish who belongs to it. Median progression-free survival in the local therapy arms was 14.2 months at the updated analysis of the trial by Gomez and colleagues and 9.7 months in the trial by Iyengar and colleagues, in which no failure occurred inside an irradiated field [5,6]. Most patients treated with curative intent therefore progressed within two years, and the absence of in-field failure indicates that the anatomic criterion admitted patients whose disease was already disseminated below the resolution of imaging. The phase II/III NRG-LU002 trial sharpened the problem: after induction systemic therapy, which was immunotherapy-based in approximately 90% of patients, adding local therapy to maintenance therapy produced no significant improvement in progression-free or overall survival [9]. Imaging cannot detect what it cannot resolve, and a computed tomography scan reports nothing about molecular disease burden, subclonal architecture or the rate at which disease is expanding.

Circulating tumor DNA (ctDNA) measures a different quantity: molecular disease burden. In the TRACERx cohort, ctDNA methods tracking a median of 200 tumor-derived mutations detected molecular residual disease after resection in patients who went on to relapse clinically, and subclones that went on to seed metastases were significantly more expanded in preoperative plasma than non-metastasizing subclones [10]. The International Association for the Study of Lung Cancer has endorsed plasma-based next-generation sequencing as an accepted route to molecular characterization in advanced NSCLC, establishing the analytical foundation on which prognostic applications are now built [11]. Throughout this review, ctDNA is treated as a quantitative marker of molecular disease burden and, after treatment, of molecular residual disease. Its established value is prognostic; it is not a validated surrogate for tumor aggressiveness or metastatic potential.

This review addresses a specific question: how should serial ctDNA measurement change the decision to operate, the timing of that operation, and what is done afterwards, in a patient who meets the anatomic definition of oligometastatic NSCLC. We examine the limits of the current definition, the analytical properties of the available assays, the evidence at each point in the surgical pathway, and the confounders that must be controlled. We then propose a framework for dynamic risk stratification, which we present as a hypothesis for prospective testing, consider how liquid biopsy applies to oncogene-driven disease and to oligorecurrence, oligoprogression and oligopersistence, and set out what remains unproven.

## 2. The Anatomic Definition and Where It Fails

The consensus definition of synchronous oligometastatic NSCLC was constructed by expert agreement, not by biological measurement. A survey of 444 European physicians preceding the consensus found that 42% accepted a maximum of three metastases, 17% accepted five or more, and 12% restricted the definition to a single lesion, while 79% considered the number of involved organs important and 71% required pathological proof of metastatic disease [12]. In the same survey, 91% of respondents completed staging with brain magnetic resonance imaging (MRI) and 98% with FDG PET-CT [12]. When 26 international experts were asked to classify ten real multidisciplinary team cases, complete agreement that a case was oligometastatic was reached for only one of the ten, and the number of distinct treatment proposals for a single case ranged from three to eight [13]. The definition is therefore a negotiated threshold rather than a measured boundary, and physicians applying it in practice do not converge.

A second limitation is biological. Oligometastatic NSCLC is a broad-spectrum condition with variable prognosis, and the behavior of the intermediate metastatic state is not explained by lesion count alone: nodal stage, tumor volume, site of distant disease, histology and performance status all modify the outcome independently [14]. In an individual patient data meta-analysis of 757 patients treated with curative intent, synchronous presentation, nodal stage and adenocarcinoma histology predicted overall survival, and five-year overall survival ranged from 13.8% for synchronous metastases with N1 or N2 disease to 47.8% for metachronous metastases [15]. A systematic review of 95 studies in which stereotactic body radiotherapy formed part of oligometastatic treatment found that the proposed characterization factors were reported so incompletely that retrospective classification under the consensus nomenclature was possible in only seven studies, and that overall survival differed significantly by primary histology, with NSCLC showing the worst outcomes of any primary site [16]. The literature on which the anatomic definition rests is therefore not internally comparable.

A third limitation is that the definition is static. It captures a single moment, whereas the clinically relevant property is trajectory. Oligoprogressive disease illustrates the point: heterogeneity in definitions persists across ongoing trials despite consensus efforts, and the risk of subsequent widespread dissemination differs substantially between subcategories, which argues for tailored rather than uniform management [17]. Surgical series make the same argument from the opposite direction. In isolated adrenal metastasis from operable NSCLC, prolonged survival after adrenalectomy depends on early T and N status of the lung primary, an R0 resection, a long disease-free interval, and confidence that no other metastatic site exists, with ipsilateral adrenal involvement carrying better outcomes than contralateral involvement because contralateral spread implies hematogenous dissemination [18]. Every one of those selection factors is a surrogate for the same unmeasured variable, namely whether the metastatic process is still confined.

The consequence is a systematic misclassification in two directions. Patients with one or two visible lesions but widespread occult dissemination are offered operations that cannot cure them. Patients with four or five lesions and a low total disease burden may be denied local therapy that could benefit them. Molecular profiling with ctDNA and circulating tumor cells is at an early stage in this population [19], but a measured quantity, rather than a count, is what is required to convert a counting rule into a risk stratification rule. Table 1 summarizes the principal definitional frameworks and their limitations, with the source of each entry.

## 3. Liquid Biopsy Analytes and Assay Architectures

Circulating tumor DNA constitutes a small and variable fraction of total cell-free DNA in plasma, and in NSCLC the median pretreatment fraction measured with an ultrasensitive hybrid-capture assay was approximately 0.1%, with levels that correlated with tumor volume [20]. Assay architecture therefore determines what a negative result means. Two designs dominate. Tumor-informed assays sequence the resected or biopsied tumor first, then track a patient-specific set of variants in plasma at very high depth. Tumor-agnostic assays interrogate a fixed gene panel without prior tissue knowledge, which permits use when tissue is unavailable but sacrifices sensitivity at low tumor fractions. Tumor-informed designs also differ in enrichment chemistry. Patient-specific multiplex PCR panels amplify variants selected from tumor sequencing [21], whereas cancer personalized profiling by deep sequencing (CAPP-Seq) applies a fixed hybrid-capture selector of recurrently mutated regions first to tumor DNA, to identify the variants of each patient, and then to plasma, to quantify them [20]. Bespoke panels track more variants per patient, a median of 200 in the most recent TRACERx analysis [10] against a median of four single-nucleotide variants with the original CAPP-Seq selector [20]; the hybrid-capture design requires no patient-specific assay construction.

The tumor-informed approach was established in NSCLC by phylogenetic ctDNA profiling of the first 100 TRACERx participants, which identified independent predictors of ctDNA release, defined the tumor volume detection limit, and detected postoperative relapse before radiological progression through blinded plasma profiling [21]. CAPP-Seq applied to 255 samples from 40 patients treated with curative intent for stage I to III lung cancer detected ctDNA in the first post-treatment blood sample in 94% of patients who subsequently recurred and preceded radiographic progression in 72% of patients by a median of 5.2 months [22]. These two studies define the upper end of a performance range that varies widely across assays and cohorts [23].

Three technical confounders constrain interpretation. First, clonal hematopoiesis generates plasma variants that are not tumor-derived. High-intensity sequencing of matched cell-free DNA and white blood cell DNA across 508 genes in 124 patients with metastatic cancer found that 53.2% of cell-free DNA mutations in patients with cancer, and 81.6% in controls without cancer, had features consistent with clonal hematopoiesis [24]. Matched leukocyte sequencing is not optional. Second, shedding is not uniform. ctDNA levels correlate with tumor volume [20]. In the first 100 TRACERx participants, ctDNA was detected before surgery in 97% of squamous cell carcinomas but in 19% of adenocarcinomas, and non-adenocarcinoma histology, lymphovascular invasion and a high Ki67 proliferation index independently predicted detection [21]. In a pooled analysis of 1425 patients, the sensitivity of plasma EGFR testing was significantly higher with extrathoracic than with intrathoracic metastases (odds ratio 5.09) [25]. A negative plasma result in a low-shedding tumor is therefore uninformative rather than reassuring, and it does not establish indolent disease. Third, anatomical compartments are not equally represented. In lung adenocarcinoma with central nervous system involvement, EGFR mutations were detected in 75% of cerebrospinal fluid samples from patients with leptomeningeal metastases vs. 36.4% of paired plasma samples, whereas the T790M resistance mutation was significantly more frequent in plasma (nine of 23) than in cerebrospinal fluid (three of 23) [26]. Plasma under-represents intracranial disease, which matters directly because brain imaging is mandatory in the consensus staging of oligometastatic disease and intracranial deposits count toward the lesion total [2]. Table 2 compares the assay architectures by variant selection, enrichment chemistry and tissue requirement; outcome data are presented in Section 6.

## 4. Before Surgery: The Baseline Sample as a Reference Measurement

The evidence that preoperative ctDNA detection carries prognostic information comes from resectable disease and does not transfer directly to oligometastatic disease. In TRACERx, which enrolled patients with resectable stage I to III NSCLC, lung adenocarcinomas without preoperative ctDNA detection had a good clinical outcome [10], and preoperative detection was associated specifically with extrathoracic recurrence [27]. These are prognostic associations. Detection depends on tumor burden and shedding, which are associated with histology, lymphovascular invasion and proliferation (Section 3), but neither detection nor its absence is a validated measure of tumor aggressiveness or metastatic competence. Patients with oligometastatic disease already carry visible metastases. With an ultrasensitive assay, ctDNA was detectable before treatment in every patient with stage II to IV NSCLC in the original CAPP-Seq series [20], although detection with less sensitive assays depends on the site of metastatic disease [25]. Detection alone therefore cannot separate patients within the oligometastatic population, and the only ctDNA correlative from a randomized oligometastatic NSCLC trial found no association between baseline ctDNA metrics and outcome [28].

The baseline sample (T1) is therefore defined by three endpoints, none of which is binary detection. First, it supplies the tumor-informed variant set against which every later sample is read. Second, it quantifies baseline molecular burden, expressed as mean variant allele fraction or as mutant molecules per milliliter of plasma, which is the reference for the kinetics measured at T2 and T3; in stage IIIA NSCLC treated with neoadjuvant chemoimmunotherapy, low pretreatment ctDNA levels were associated with longer progression-free survival (hazard ratio 0.20) and overall survival (hazard ratio 0.07) [29]. Third, it establishes whether the tumor sheds, which determines whether a later negative result can be interpreted at all.

In synchronous oligometastatic disease, the baseline assay can be built from tissue that the consensus workup already requires, because histological confirmation of a metastatic site is mandatory [2]. We recommend four steps. First, sequence the primary tumor and the biopsied metastasis together with matched white blood cells, so that clonal hematopoiesis variants are excluded [24] and variants shared by both sites can be identified. Second, build the panel predominantly from clonal variants. In the first 100 TRACERx participants, panels targeting a median of 11 clonal and six subclonal variants detected clonal variants in every ctDNA-positive patient, with a median of 94% of targeted clonal variants detected against 27% of subclonal variants [21]; variants shared by the primary and a metastasis are truncal and are carried by every deposit that descends from the same clone. Third, draw and bank the baseline plasma before any systemic therapy, because panel construction takes weeks and the reference value cannot be recovered once induction has begun. Fourth, report the burden as mutant molecules per milliliter of plasma as well as variant allele fraction, record shedding status explicitly, add digital PCR of the driver variant in oncogene-driven disease (Section 8.1), and consider cerebrospinal fluid when oligometastatic status depends on intracranial disease [26]. None of these steps separates synchronous oligometastatic from more disseminated disease at baseline, where ctDNA is detectable in almost every patient with an ultrasensitive assay [20]; if ctDNA can make that distinction, it will do so through the trajectory from T1 to T3 (Section 5).

Subclonal architecture is an exploratory endpoint at T1, not a decision endpoint. Measurement of subclone cancer cell fractions in preoperative plasma showed that subclones seeding future metastases were significantly more expanded than non-metastatic subclones, and non-invasive tracking of subclonal architecture identified patients with polyclonal metastatic dissemination, which carried a poor clinical outcome [10]. If polyclonal dissemination were demonstrated in oligometastatic disease, it would be difficult to reconcile with an arrested intermediate state, because removing the visible deposits would address only part of the disease. These observations come from resectable disease and have not been tested in oligometastatic cohorts.

**Figure 1:**
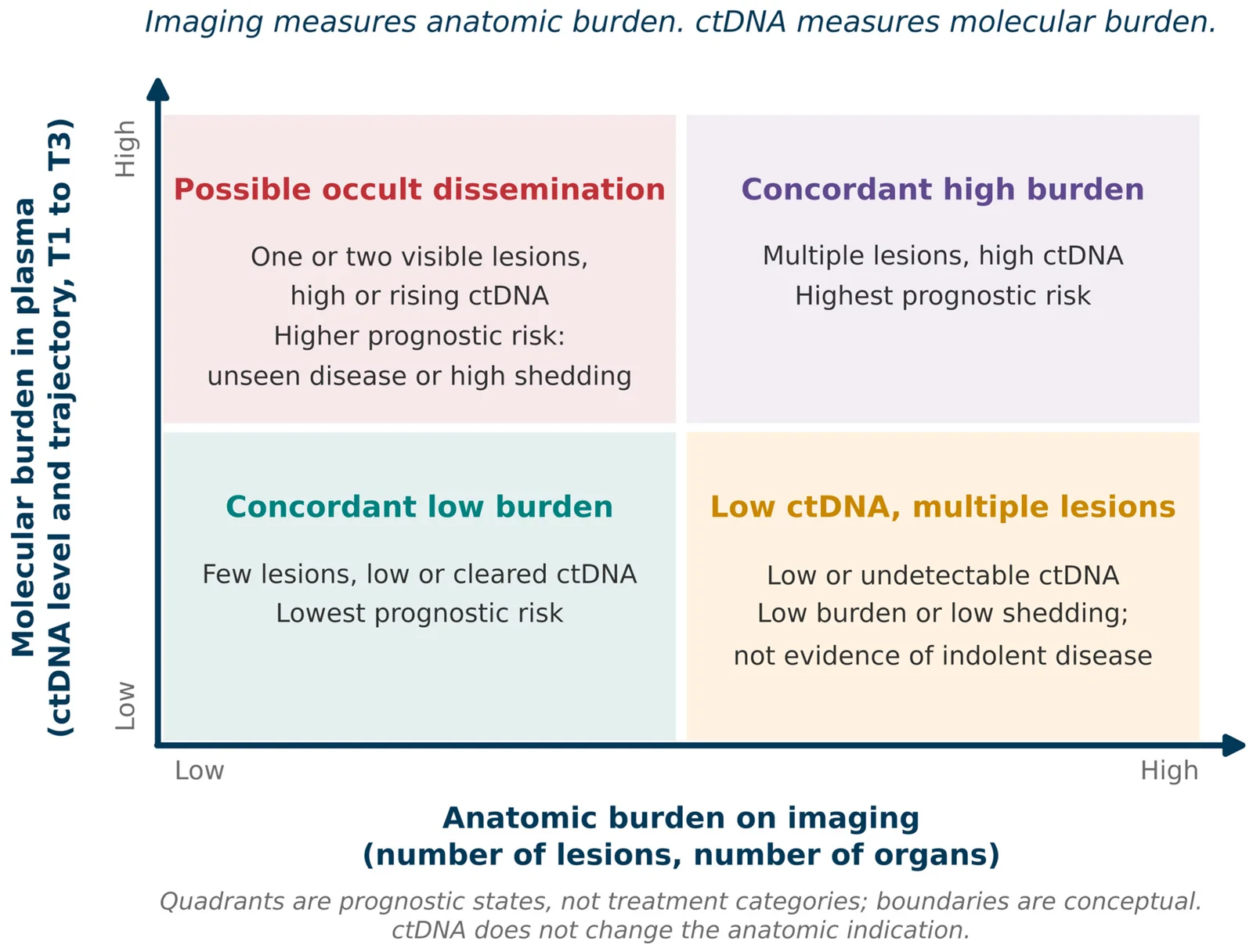
Two-axis framework for oligometastatic NSCLC. The horizontal axis represents anatomic burden as measured by imaging, the basis of the current consensus definition. The vertical axis represents molecular burden and its trajectory across the baseline, on-induction and preoperative samples, not a single baseline value. The four quadrants describe prognostic states, not validated treatment categories. Discordant quadrants are invisible to anatomic staging alone, and low or undetectable ctDNA reflects low burden or low shedding; it does not establish indolent disease. Quadrant boundaries are conceptual and are not defined by validated thresholds.

Figure 1 places these measurements on two axes. Imaging defines an anatomic axis and ctDNA defines an orthogonal axis of molecular disease burden, read as the trajectory from T1 through T3 rather than as a single baseline value. The quadrants describe prognostic states, not validated treatment categories, and their boundaries are conceptual. Concordant low anatomic and molecular burden identifies the patient at lowest prognostic risk, and concordant high burden the patient at highest risk. Discordance in which one or two visible lesions coexist with high or rising ctDNA may indicate a molecular burden that imaging has not accounted for, consistent with occult polymetastatic disease; it can also reflect a high-shedding visible lesion and requires restaging before it is interpreted as dissemination. Discordance in the other direction, with multiple lesions and low or undetectable ctDNA, reflects either a low molecular burden or a low-shedding tumor; it does not establish indolent disease, and it requires histological confirmation before local therapy is withheld. No quadrant changes an anatomic indication for surgery.

Two cautions apply. Undetectable preoperative ctDNA does not prove absence of micrometastatic disease, because detection depends on shedding and on tumor volume, and small or low-shedding tumors fall below the limit of detection. An undetectable result therefore indicates a molecular burden below the detection limit of the assay, not indolent disease. Detectable preoperative ctDNA is a continuous rather than a binary variable, and no level of molecular burden has been shown in any prospective trial to justify modifying an anatomic indication for surgery.

## 5. During Induction: ctDNA Kinetics as a Measure of Systemic Response

Current practice in oligometastatic NSCLC already uses time as a selection instrument. Both positive randomized trials required an interval of systemic therapy without progression before local consolidative therapy was delivered [4,6]. That interval functions as a clinical test: patients whose disease progresses during induction are removed from the local therapy pathway. The hypothesis examined here is that serial ctDNA reads the same test earlier and with greater sensitivity than imaging.

Direct evidence in the oligometastatic setting is limited but consistent in direction. In a translational analysis of the randomized trial by Gomez and colleagues, circulating tumor DNA from 31 patients was analyzed by next-generation sequencing across a 1021-gene panel. At early follow-up, local consolidative therapy was associated with fewer detected mutations (median 2, interquartile range 1 to 6) than maintenance therapy or observation (median 6, interquartile range 4 to 18), and with lower average variant allele frequency (median 0.006 vs. 0.011). Among six patients with serial analysis, a rise in the ctDNA mutation burden preceded clinical progression by 6.7 months [28]. This remains the only ctDNA correlative embedded in a randomized oligometastatic NSCLC trial. Its analyses were exploratory and not corrected for multiple testing [28], but they show that local therapy was associated with lower measurable molecular burden and that a molecular rise preceded clinical progression.

**Figure 2:**
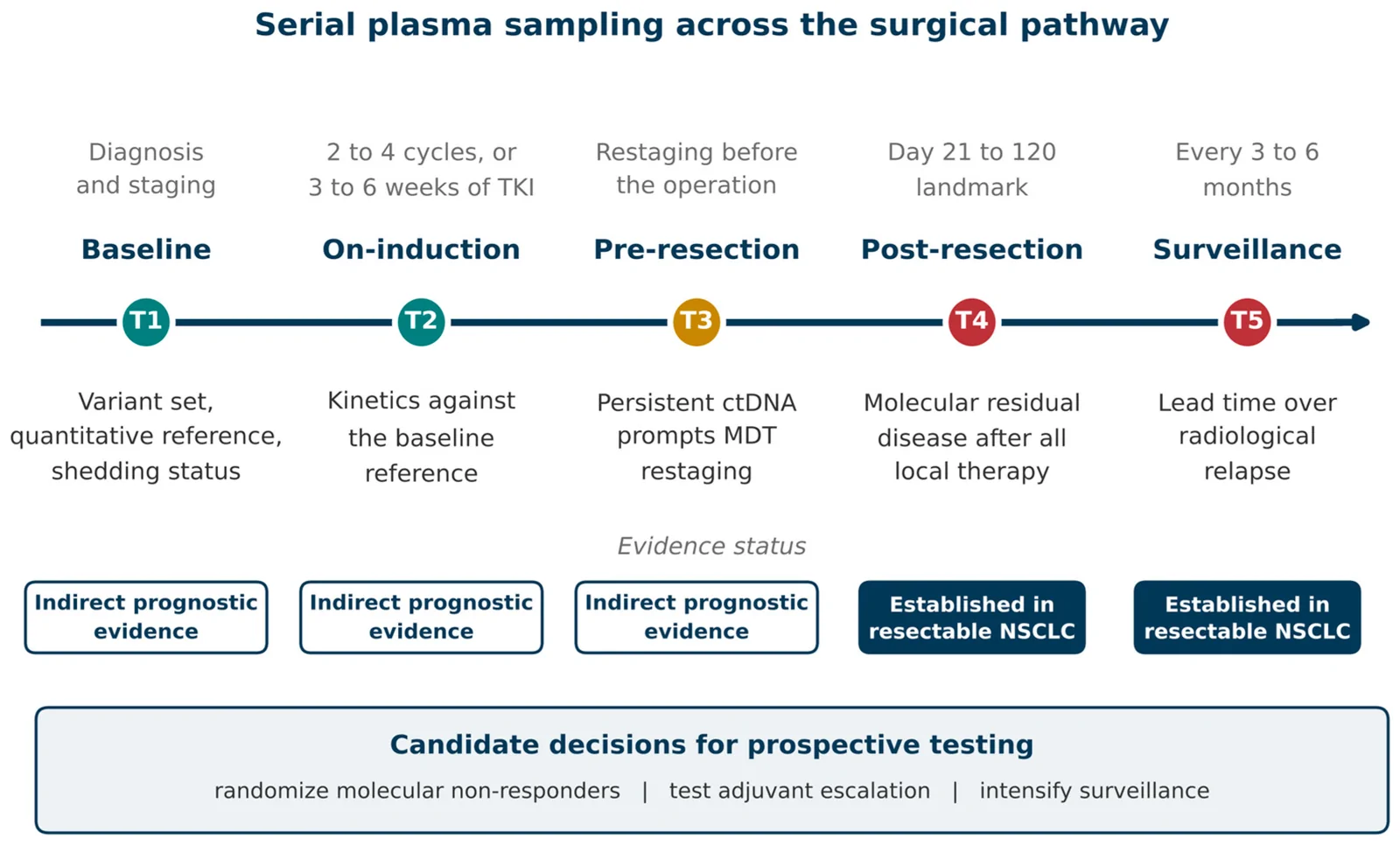
Serial plasma sampling across the surgical pathway in oligometastatic NSCLC. Sampling at diagnosis (T1) establishes a tumor-informed panel, a quantitative baseline reference and the shedding status of the tumor. Sampling during induction (T2) and at restaging (T3) measures kinetics against that reference. Landmark postoperative sampling (T4) identifies molecular residual disease after all local therapy is complete. Surveillance sampling (T5) provides lead time over radiological relapse. The evidence status of each point is shown beneath it: postoperative and surveillance sampling (T4, T5) are established prognostic markers in resectable NSCLC; baseline, on-induction and preoperative sampling (T1 to T3) rest on indirect prognostic evidence from neoadjuvant or advanced NSCLC. In oncogene-driven disease, T2 can be drawn after 3 to 6 weeks of targeted therapy (Section 8.1). At each point, plasma results inform but do not override anatomic and histological findings; the decisions listed beneath the timeline are hypotheses for prospective testing.

Evidence that induction-phase ctDNA carries prognostic weight before resection comes from resectable disease. In the single-arm NADIM trial of neoadjuvant nivolumab plus chemotherapy for stage IIIA NSCLC, undetectable ctDNA after neoadjuvant treatment was associated with longer progression-free survival (hazard ratio 0.26) and overall survival (hazard ratio 0.04), and ctDNA after induction predicted overall survival better than radiological response (C-index 0.82 vs. 0.72) [29]. In CheckMate 816, neoadjuvant nivolumab plus platinum-based chemotherapy produced a pathological complete response in 24.0% of patients vs. 2.2% with chemotherapy alone, and median event-free survival of 31.6 months vs. 20.8 months, with 83.2% of the experimental group proceeding to surgery [30]. In NADIM II, perioperative nivolumab plus chemotherapy in resectable stage IIIA or IIIB disease produced a pathological complete response in 37% vs. 7%, surgery in 93% vs. 69%, and 24-month overall survival of 85.0% vs. 63.6% [31]. CheckMate 816 and NADIM II established that a period of systemic therapy before resection is safe, that it does not impair operability, and that the depth of response measured after induction carries prognostic weight. Plasma sampling during that same interval requires no additional procedure. Figure 2 sets out the five sampling points that structure this approach across the surgical pathway. T1 is drawn at diagnosis, before any systemic therapy; T2 during induction, after two to four cycles of chemotherapy-based treatment or 3 to 6 weeks of targeted therapy; T3 at restaging before local therapy; T4 between 21 and 120 days after resection; and T5 every 3 to 6 months thereafter.

The hypothesis that follows is testable but untested. When ctDNA clears or falls during induction, with radiological stability or response, the systemic component of the disease may be controlled and the visible deposits may be the dominant remaining burden; resection of all sites would then have its greatest chance of cure. When ctDNA persists or rises despite radiological stability, systemic disease may remain active that resection of the visible sites will not address, or the signal may originate from the visible deposits that local therapy would remove (Section 7). Both statements are extrapolations from prognostic data, neither has been tested in oligometastatic NSCLC, and no validated threshold separates the categories. In a pooled analysis of 200 patients from five trials of immune checkpoint inhibitors in advanced NSCLC, on-treatment reductions in ctDNA were associated with longer overall and progression-free survival, but the five trials used different assays and required post hoc harmonization of maximum variant allele frequency [32]; in NADIM, the discriminating endpoint was complete clearance [29]. We therefore use clearance, decline, and persistence or rise as descriptive categories, not as decision thresholds.

## 6. After Resection: Molecular Residual Disease

The postoperative landmark is the point at which liquid biopsy is closest to clinical implementation. In TRACERx, plasma collected within 120 days of surgery detected ctDNA in 25% of patients, and that group included 49% of all patients who went on to relapse clinically. Surveillance sampling every 3 to 6 months identified impending relapse in a further 20% of patients who had been negative at the landmark [10]. In the CAPP-Seq series described in Section 3, 53% of patients harbored mutation profiles associated with responsiveness to tyrosine kinase inhibitors or immune checkpoint blockade [22].

Three consequences follow for oligometastatic disease specifically. First, a positive landmark result after resection of all known sites indicates molecular residual disease and a high risk of relapse, irrespective of R0 status on pathology. Second, the median lead time of approximately 5 months defines a window in which escalation of systemic therapy could be tested while disease burden is minimal. Third, a negative landmark result in a patient with a demonstrably shedding tumor, meaning one that was ctDNA-positive at baseline, identifies the lowest risk of relapse that current assays can define; it does not exclude micrometastatic disease below the limit of detection. The requirement that the tumor was positive at baseline is essential, because a negative postoperative result in a tumor that never shed is uninterpretable. Table 3 collates the evidence linking ctDNA to the outcome at each point in the pathway.

## 7. A Framework for Dynamic Risk Stratification: A Hypothesis for Prospective Testing

**Figure 3:**
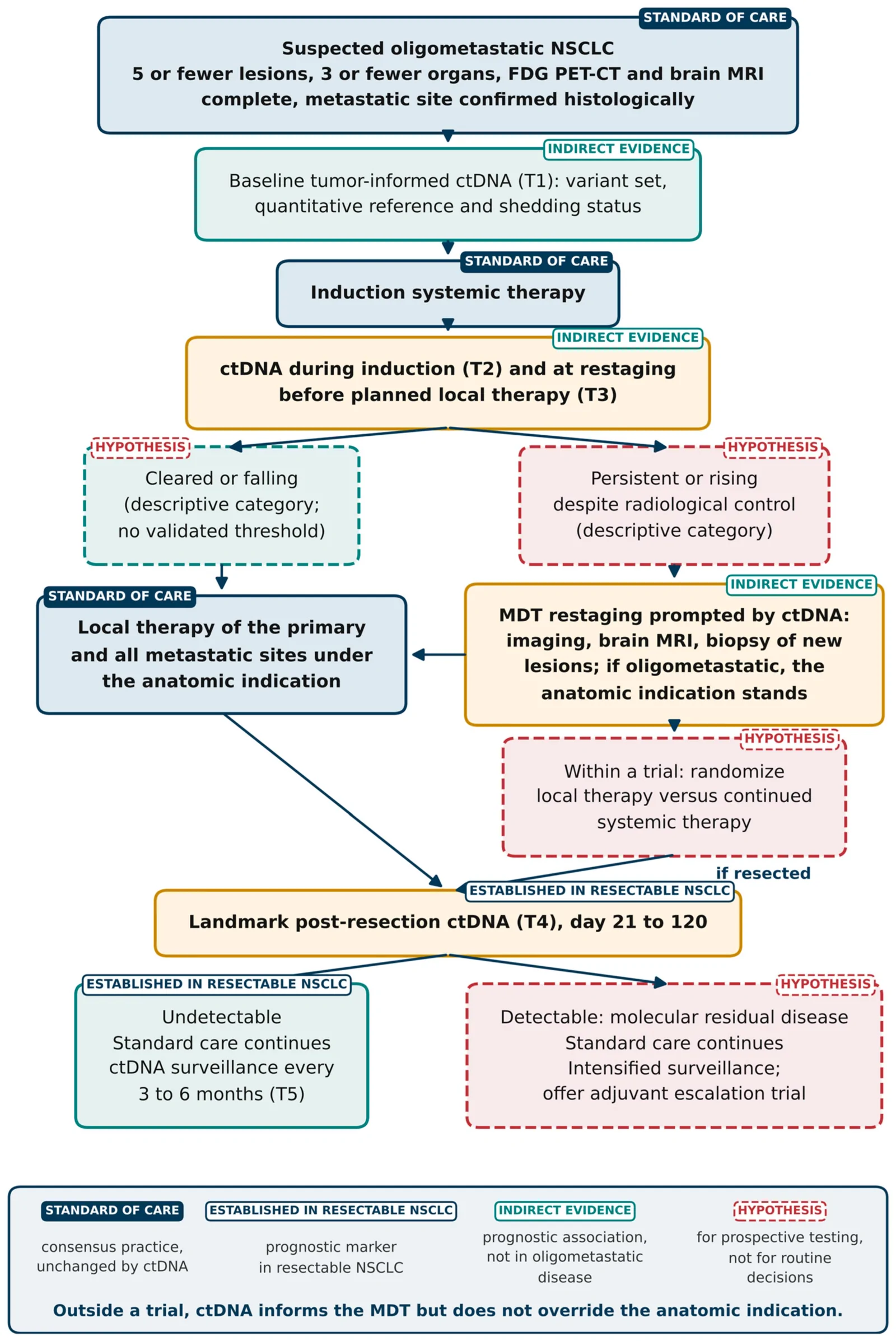
Proposed framework for ctDNA-informed surgical decision-making in oligometastatic NSCLC, presented as a hypothesis for prospective testing. Anatomic eligibility is established first and is not modified by plasma findings. A tumor-informed assay is constructed at baseline. During induction, clearance or decline of ctDNA is consistent with systemic control, and local therapy of all sites proceeds under the anatomic indication. Persistent or rising ctDNA prompts multidisciplinary restaging; if restaging confirms oligometastatic disease, the anatomic indication stands and the patient proceeds to the same local therapy, and only within a trial would these patients be randomized between local therapy and continued systemic therapy. After resection, standard care continues in all patients; landmark sampling identifies molecular residual disease and could inform surveillance intensity and eligibility for adjuvant escalation trials. Labels mark the evidence status of each node using the four levels of Table 4: standard of care (consensus practice, unchanged by ctDNA), established prognostic marker in resectable NSCLC, indirect prognostic evidence, and hypothesis (dashed outline; for prospective testing, not for routine decisions). No validated threshold defines the response categories.

Figure 3 sets out a framework that integrates the preceding evidence. We present it as a research hypothesis, not as a clinical algorithm: none of its plasma-triggered actions has been validated prospectively, and none should change an operative indication outside a clinical trial. A recent review of local therapy in oligometastatic NSCLC reached the same conclusion, placing ctDNA in trial enrichment and post-treatment surveillance research rather than in routine decision rules for offering or withholding stereotactic radiotherapy [33]. Entry requires the full anatomic workup mandated by consensus, namely five or fewer lesions across three or fewer organs, complete FDG PET-CT and brain magnetic resonance imaging, and histological confirmation of a metastatic site [2]. Liquid biopsy is added to that workup, not substituted for any part of it. Figure 3 and Table 4 classify each element of the framework by the same four levels of evidence: standard of care, established prognostic marker in resectable NSCLC, indirect prognostic evidence, and hypothesis.

Four principles govern the framework. First, ctDNA modifies probability but does not establish diagnosis: no patient should be denied a potentially curative operation on the basis of a plasma result alone, and no positive plasma result should trigger treatment without radiological or histological correlation. Second, kinetics outrank single measurements, because a single detectable result reflects shedding as much as the burden, whereas the trajectory across two or more time points reflects the response. Third, the assay must be tumor-informed and accompanied by matched leukocyte sequencing, otherwise more than half of the cell-free DNA variants detected may derive from clonal hematopoiesis [24]. Fourth, patients with intracranial disease require compartment-appropriate assessment, because plasma systematically under-represents central nervous system deposits [26].

The framework operates in two modes. Outside a randomized trial, it changes no operative indication. A persistent or rising ctDNA level at restaging prompts the multidisciplinary team to look for the disease that plasma suggests and imaging has not shown, through repeat cross-sectional imaging, brain MRI and biopsy of any new suspicious lesion; if restaging confirms oligometastatic disease, the anatomic indication for resection stands. A stable or slightly rising level in that patient may originate from the visible deposits that local therapy is designed to remove: local consolidative therapy was associated with lower measurable ctDNA burden than maintenance therapy or observation in the only randomized oligometastatic correlative [28], and a completed non-randomized phase II study (NCT05429320) delivered local ablative therapy specifically to patients whose mean variant allele fraction fell by less than 50% during first-line systemic therapy without radiographic progression. Within a clinical trial, the framework generates three testable hypotheses: that molecular non-responders gain less from resection than from continued systemic therapy or, conversely, that resection is what lowers their molecular burden; that patients with four or five lesions and molecular clearance gain from resection as much as patients with fewer lesions; and that adjuvant escalation in landmark-positive patients improves survival. Section 10 sets out the designs that would test each.

## 8. Liquid Biopsy in Oncogene-Driven Disease and Other Oligometastatic States

### 8.1. Oncogene-Driven Disease

Patients with EGFR- or ALK-driven oligometastatic disease differ from driver-negative patients in four ways that change how plasma is used. First, plasma identifies the driver itself. In the BFAST trial, hybrid-capture plasma sequencing returned a result in 98.6% of 2219 patients with advanced NSCLC and identified ALK-positive disease in 5.4%, and alectinib produced an objective response rate of 87.4% in that plasma-selected cohort [36]. Plasma genotyping at T1 therefore shortens the interval to the targeted induction therapy on which every later decision depends. Second, the driver is a truncal alteration, so a single-variant digital PCR assay can quantify response without a bespoke panel (Table 2). In FLAURA, patients without detectable plasma EGFR mutations at baseline had longer progression-free survival (hazard ratio 0.54), and clearance by week 3 of osimertinib was associated with longer median progression-free survival than persistence (19.8 vs. 11.3 months) [34]. In oncogene-driven disease the T2 sample can therefore be drawn after 3 to 6 weeks, at the time points assessed in FLAURA, rather than after two to four cycles. Third, the purpose of local therapy differs. In EGFR-mutant disease, consolidation aims to extend the benefit of an effective inhibitor rather than to replace systemic control, and in the randomized phase II NorthStar trial, as summarized in a recent review, osimertinib plus local consolidative therapy prolonged median progression-free survival compared with osimertinib alone (25.3 vs. 17.5 months) [33].

Fourth, and most important for surgical decisions, targeted therapy suppresses ctDNA without necessarily eradicating disease. In a post hoc analysis of 220 patients from ADAURA, tumor-informed molecular residual disease preceded imaging relapse by a median of 4.7 months, but in the osimertinib group 19 of 28 molecular or clinical relapse events (68%) occurred after the drug was stopped [35]. A negative landmark result in a patient who continues a tyrosine kinase inhibitor after resection may therefore reflect suppression by the drug rather than clearance of residual disease; the landmark is most informative off treatment or during surveillance after treatment ends. Plasma clearance on an inhibitor likewise does not show that the residual deposits are sterilized, which is the rationale for consolidating them. The framework therefore applies to oncogene-driven disease with three modifications: genotyping and baseline quantification of the driver at T1, early driver-variant kinetics at T2, and interpretation of postoperative results in the light of continuing targeted therapy. Whether these modifications identify the patients who gain most from consolidation is untested. A single-arm phase II trial of local therapy to the primary tumor after osimertinib plus chemotherapy (NCT07073365) measures ctDNA before and after induction as an exploratory endpoint [37], and in the randomized trials listed in Table 5 the role of ctDNA in oncogene-driven disease is limited to exploratory and resistance analyses.

### 8.2. Oligorecurrence, Oligoprogression and Oligopersistence

The framework in Section 7 addresses synchronous disease, but the consensus nomenclature separates repeat and induced oligometastatic states, and the role of ctDNA differs in each [3]. Oligorecurrence, the metachronous relapse after curative-intent treatment, is the state in which the evidence is strongest, because it is the clinical continuation of the postoperative surveillance described in Section 6. Surveillance ctDNA after resection identifies relapse before imaging [10,22], and metachronous presentation carries a better prognosis than synchronous disease [15]. A rising ctDNA level during surveillance could therefore prompt targeted imaging while recurrence is still limited and amenable to local therapy. Whether earlier detection of oligorecurrence improves survival, rather than advancing the date of diagnosis, has not been tested.

In oligoprogression, a few sites progress while the remaining disease stays controlled, and ctDNA serves a different purpose: it characterizes the mechanism of resistance. In FLAURA, next-generation sequencing of paired plasma samples at progression on first-line osimertinib identified MET amplification in 16% and EGFR C797S mutations in 6% of patients with paired samples [38]. A resistance mechanism detected in plasma may favor a systemic switch, whereas progression without a new plasma alteration is compatible with, but does not establish, focal escape that local therapy can address while the effective agent is continued; it can also reflect non-genetic resistance [38] or low shedding. Plasma does not replace biopsy of the progressing site, because a single progressing deposit may shed too little DNA to be represented and intracranial progression is under-represented in plasma [25,26]. The HALT trial (NCT03256981) measures resistant subclones in ctDNA as a secondary endpoint, and the single-arm CURB-TKI study (NCT06813664), not yet recruiting, will use ctDNA after stereotactic radiotherapy to tailor subsequent targeted therapy.

Oligopersistence, the residual disease at a few sites after a response to systemic therapy, is the state that the induction-phase sampling of Section 5 addresses, and it is the setting of several consolidation trials in oncogene-driven disease, including NorthStar and TARGET-01 [33]. Persistent plasma driver mutations after targeted induction identify patients at higher risk of progression (Section 8.1). Whether the residual deposits in those patients are the source of the persisting signal, and therefore the right target for consolidation, is the question that the induction-phase hypothesis of Section 5 and Section 7 poses.

## 9. Limitations and Unresolved Problems

No randomized trial has yet used ctDNA to allocate patients to or from surgery in oligometastatic NSCLC. The framework proposed here is constructed from prognostic associations, and prognostic value does not establish predictive value. A biomarker that identifies patients destined to relapse does not by itself demonstrate that withholding surgery from those patients improves outcome, and it is possible that molecular non-responders derive benefit from cytoreduction that current evidence cannot detect. A related limitation is interpretive. ctDNA quantifies the burden of disease that sheds into plasma, and its established value is prognostic; it is not a validated measure of tumor aggressiveness or metastatic potential, so neither a high nor an undetectable level can be read as a statement about tumor biology.

The threshold definition is unresolved. The evidence supports a binary detected or undetected reading at the postoperative landmark, and supports directional interpretation of kinetics, but no validated cut-off exists for variant allele fraction, for the magnitude of decline that should count as a molecular response, or for the interval after surgery at which the landmark sample should be drawn. The 120-day window used in TRACERx and the 21-day lower bound reflect assay logistics and postoperative cell-free DNA release rather than an optimized decision point [10]. The response categories used in Section 5 and Section 7 are therefore descriptive and unvalidated.

Assay heterogeneity limits generalization. Across published studies of molecular residual disease after curative-intent treatment of NSCLC, sensitivity for relapse at the first post-treatment time point ranged from 36% to 100% and specificity from 71% to 100% [23], and results obtained with one architecture cannot be transferred to another. Reimbursement and turnaround time constrain deployment: a bespoke panel requires tissue, sequencing and design time that must fit inside the interval between diagnosis and the start of induction therapy.

Cost is a separate constraint. The framework requires tissue sequencing, construction of a tumor-informed panel and up to five plasma assays per patient, and the cost-effectiveness of serial ctDNA monitoring remains unresolved across solid tumors [39]. We found no economic evaluation in oligometastatic NSCLC. The closest model, a simulation of ctDNA-guided treatment switching in advanced NSCLC, estimated a survival gain of 4.0 weeks at an additional cost of USD 3975 per patient, an incremental cost-effectiveness ratio of USD 87,941 per quality-adjusted life year, and a 66% probability of cost-effectiveness at a threshold of USD 150,000 per quality-adjusted life year [40]. Serial testing that does not change management adds cost without benefit, which is a further reason to confine it to prospective studies until its effect on treatment decisions is known.

Selection bias affects every surgical series in this field. Reporting of the consensus characterization factors is so incomplete that classification of published cohorts under the current nomenclature was possible in seven of 95 studies [16], and the surgical literature on metastasectomy consists largely of retrospective, uncontrolled series in which assignment to operation was determined by unrecorded clinician judgment [18]. Adding a biomarker to a study with this structure does not correct it. Finally, the definitional instability documented above propagates into any biomarker study built on it. If 26 experts reach full agreement on whether a case is oligometastatic in one of 10 instances [13], then trials enrolling on that definition are enrolling heterogeneous populations, and a biomarker validated in one such population may not transfer to another.

## 10. Future Directions

Three trial designs would resolve the principal uncertainties. A biomarker-stratified randomized trial should enroll anatomically defined oligometastatic patients, measure ctDNA after induction therapy, and randomize molecular non-responders to local consolidative therapy or to continued systemic therapy alone, with overall survival as the primary endpoint. This is the only design that converts prognostic association into predictive evidence, and it tests the framework where it matters, which is in the group currently offered operations of uncertain value. The negative NRG-LU002 result [9] makes this design more pressing, because the benefit of local therapy can no longer be assumed once induction is immunotherapy-based, and a biomarker that identifies the patients who still benefit is the most direct way to reconcile that result with the earlier phase II trials.

A postoperative escalation trial should randomize patients who are ctDNA-positive at the landmark after complete local therapy to intensified systemic treatment or to standard surveillance. Given a 25% landmark positivity rate and a lead time of approximately 5 months, such a trial is feasible within existing multicenter networks [10,22].

An observational registry with mandatory reporting of the consensus characterization factors, paired plasma sampling at all five defined time points, and prespecified assay architecture would address the incomparability of the current literature [3,16]. Within that structure, the specific questions that require answers are the shedding threshold below which a negative result is uninterpretable, the role of cerebrospinal fluid sampling in patients whose oligometastatic status depends on intracranial disease, and whether subclonal architecture in preoperative plasma predicts polyclonal dissemination reliably enough to be used prospectively [10,26].

No ongoing randomized trial of local therapy in oligometastatic NSCLC uses ctDNA to select, stratify or allocate patients (Table 5). In the randomized trials cataloged in a recent review [33] and identified in a registry search, ctDNA appears only as a secondary or exploratory endpoint, when it appears at all. The phase III OliGRAIL trial (NCT06840782) measures ctDNA clearance at randomization, week 6 and month 4 and includes a cost-utility analysis. NRG-LU002 (NCT03137771), whose primary results have been presented and are summarized in a recent review [9], lists the correlation between ctDNA and clinical outcome among its objectives; if that analysis tests the interaction between post-induction ctDNA and treatment arm, it would provide a randomized test of whether ctDNA identifies the patients who benefit from local therapy. Only non-randomized studies use ctDNA to decide who receives local therapy: NCT05429320 delivered it for a non-responding variant allele fraction, and NCT07410611, not yet recruiting, states that dynamic ctDNA monitoring will guide radiotherapy combined with befotertinib in EGFR-mutant disease, without specifying the rule in its registry record. The biomarker-stratified randomized trial proposed above has no current equivalent.

## 11. Conclusions

Oligometastatic NSCLC is defined by counting lesions, and counting cannot answer the question that determines the surgical benefit. Randomized phase II evidence shows that local consolidative therapy extended median overall survival from 17.0 to 41.2 months in one trial of anatomically selected patients, yet most of those patients progressed within two years and the phase II/III NRG-LU002 trial was negative, which means the selection is imprecise. Circulating tumor DNA measures molecular burden, its response to systemic therapy and residual disease after resection, with a lead time over imaging of approximately 5 months. Its established value is prognostic.

The evidence is sufficient to justify collecting serial plasma samples from every candidate for oligometastatic resection within prospective studies and registries, and to justify reporting persistent or rising ctDNA to the multidisciplinary team as a prompt for restaging. In oncogene-driven disease, plasma results must also be read in the light of continuing targeted therapy, which suppresses ctDNA without necessarily eradicating disease. It is not sufficient to permit a plasma result to defer or overrule an anatomic indication for surgery. Closing that gap requires biomarker-stratified randomized trials in which ctDNA allocates treatment rather than merely describing it.

## Figures and Tables

**Table 1:** Principal frameworks defining oligometastatic disease in NSCLC and their limitations for surgical selection. Sources are cited in the first column of each row and, for individual figures, in the corresponding cell.

Framework	Core Criterion	Staging Requirement	Limitation for Surgical Selection
Hellman and Weichselbaum, 1995 [1]	Conceptual intermediate state between localized and disseminated disease	None specified	Conceptual only; no operational threshold
TNM eighth edition (M1b) [13]	Single extrathoracic metastasis in a single organ	Conventional staging	Excludes most patients treated with curative intent in randomized trials, which enrolled up to three [4] or five [6,7] metastases
EORTC consensus, 2019 [2]	5 or fewer lesions, 3 or fewer organs; mediastinal nodes not counted	FDG PET-CT and brain imaging mandatory; biopsy of a solitary metastasis mandated	Threshold set by expert agreement, not by outcome data; static single time point
ESTRO and EORTC nomenclature, 2020 [3]	Genuine, induced, repeat and de novo states; oligorecurrence, oligoprogression, oligopersistence	Requires full treatment history	No biomarker anchors any category; retrospective application feasible in 7 of 95 published studies [16]
Physician surveys, 2019 [12,13]	Maximum number of metastases accepted: 3 or fewer (42%), 5 or more (17%), a single lesion (12%) [12]	Brain MRI used by 91% and FDG PET-CT by 98% of respondents [12]	Complete expert agreement in 1 of 10 cases; 3 to 8 treatment proposals per case [13]

**Table 2:** Circulating tumor DNA assay architectures relevant to surgical decision-making in oligometastatic NSCLC, classified by variant selection and enrichment chemistry.

Architecture	Variant Selection and Enrichment Chemistry	Tissue Requirement	Principal Limitation	Role in the Surgical Pathway
Tumor-informed, bespoke panel [10,21]	Patient-specific variants selected from tumor sequencing; patient-specific multiplex PCR [21]	Required; panel built after tissue sequencing	Panel design time; variants absent from the sequenced tissue are not tracked	Baseline reference, induction kinetics and postoperative molecular residual disease
Tumor-informed, fixed hybrid-capture panel (CAPP-Seq) [20,22]	Fixed selector of recurrently mutated regions applied first to tumor DNA and then to plasma; hybrid capture with deep sequencing [20]	Required to identify the variants of each patient	Fewer variants tracked per patient than bespoke panels [20]	Molecular residual disease and surveillance without patient-specific assay construction
Tumor-agnostic, fixed gene panel [11,24]	Variants called de novo in plasma across a fixed gene panel	Not required	Clonal hematopoiesis variants indistinguishable from tumor variants without matched leukocyte sequencing [24]	Genotyping for treatment selection; not designed to exclude residual disease
Digital PCR [26]	One predefined variant quantified in partitioned PCR reactions	Requires a known driver variant	Single variant; no information on heterogeneity	Monitoring a known driver such as EGFR, including in cerebrospinal fluid

All sequencing-based designs require matched white blood cell sequencing to exclude clonal hematopoiesis variants [24]. Any design can be applied to cerebrospinal fluid when intracranial disease determines oligometastatic status [26]. Circulating tumor cells have no current role in operative selection [19]. Outcome data for these assays are summarized in Section 6.

**Table 3:** Evidence linking circulating tumor DNA to clinical outcome across the local therapy pathway in NSCLC.

Study and Setting	Population	ctDNA Endpoint	Principal Finding
Abbosh et al., 2017 [21]; TRACERx 100, resected NSCLC	First 100 TRACERx participants	Phylogenetic tracking in postoperative plasma	Blinded postoperative profiling identified patients highly likely to relapse and tracked the subclonal nature of metastasis
Chaudhuri et al., 2017 [22]; stage I to III lung cancer treated with curative intent	40 patients, 255 samples	Molecular residual disease after treatment	ctDNA present in the first post-treatment sample in 94% of patients who recurred; median 5.2 months of lead time over imaging
Abbosh et al., 2023 [10]; TRACERx, resected NSCLC	197 patients, 1069 plasma samples	Landmark detection within 120 days of surgery	ctDNA detected in 25% of patients, comprising 49% of all clinical relapses; surveillance identified a further 20% of landmark-negative relapses
Karasaki et al., 2023 [27]; TRACERx lung adenocarcinoma	248 tumors, 805 regions	Preoperative ctDNA detection	Preoperative detection associated specifically with extrathoracic recurrence
Tang et al., 2020 [28]; randomized oligometastatic NSCLC (NCT01725165)	31 patients with ctDNA analysis	Mutation number and variant allele frequency after local therapy	Local consolidative therapy associated with fewer detected mutations (median 2 vs. 6) and lower variant allele frequency (0.006 vs. 0.011) than maintenance therapy or observation; ctDNA rise preceded progression by 6.7 months
Provencio et al., 2022 [29]; NADIM, stage IIIA NSCLC, neoadjuvant chemoimmunotherapy	Single-arm phase II trial	Pretreatment level and detectability after induction	Low pretreatment ctDNA and undetectable ctDNA after induction associated with longer progression-free and overall survival; ctDNA after induction outperformed radiological response for predicting overall survival

**Table 4:** Evidence status of each element of the proposed framework.

Element of the Framework	Evidence Status	Basis	Required Before Clinical Use
Anatomic eligibility: 5 or fewer lesions, 3 or fewer organs, FDG PET-CT, brain MRI and histological confirmation	Standard of care	Expert consensus [2]; eligibility criteria of the randomized phase II trials [4,6,7]	Not modified by ctDNA in this framework
Postoperative landmark ctDNA (T4) as a marker of molecular residual disease	Established prognostic marker in resectable NSCLC	Landmark cohorts in resected NSCLC (Table 3) [10,22]; sensitivity varies widely across assays [23]	Validation in oligometastatic NSCLC; evidence that treatment guided by a positive result improves survival
Surveillance ctDNA (T5) as an early signal of relapse	Established prognostic marker in resectable NSCLC; exploratory in oligometastatic NSCLC	Lead time over imaging in resected NSCLC (Table 3) [22]; one exploratory series of 6 patients with oligometastatic disease [28]	Evidence that earlier treatment of molecular relapse improves outcome
Baseline ctDNA level (T1) as a prognostic reference	Indirect prognostic evidence	Associated with survival in stage IIIA disease treated with neoadjuvant chemoimmunotherapy (Table 3) [29]; no association with outcome in the only oligometastatic correlative [28]	Prospective oligometastatic cohorts with prespecified assays
ctDNA kinetics during induction (T2, T3) as a measure of systemic response	Indirect prognostic evidence	Clearance after neoadjuvant chemoimmunotherapy [29] and on-treatment decline in advanced NSCLC [32] associated with survival	A validated response threshold; testing in oligometastatic NSCLC
Persistent or rising ctDNA at T3 prompts multidisciplinary restaging (restaging itself is standard of care)	Indirect prognostic evidence	Persistent ctDNA after neoadjuvant chemoimmunotherapy [29] and on immune checkpoint inhibitors [32] associated with shorter survival	Evidence that ctDNA-prompted restaging detects disease that imaging missed
Driver-variant kinetics and postoperative ctDNA in oncogene-driven disease (T2 to T4)	Indirect prognostic evidence	Early plasma EGFR clearance on osimertinib associated with longer progression-free survival [34]; molecular relapse after adjuvant osimertinib occurred mostly after the drug was stopped [35]	Testing in oligometastatic disease; landmark sampling off targeted therapy
Persistent or rising ctDNA identifies patients who gain less from resection	Hypothesis	Prognostic association only; prognostic value does not establish predictive value	Biomarker-stratified randomized trial (Section 10)
Patients with 4 or 5 lesions and ctDNA clearance gain from resection as much as patients with fewer lesions	Hypothesis	No direct data	Registry with paired sampling and a stratified trial (Section 10)
Adjuvant escalation in landmark-positive patients improves survival	Hypothesis	No data in oligometastatic NSCLC	Randomized escalation trial (Section 10)

T1 to T5, plasma sampling points defined in Figure 2. Standard of care, consensus practice that ctDNA does not modify; established prognostic marker, association with relapse replicated in resectable NSCLC; indirect prognostic evidence, association shown in neoadjuvant or advanced NSCLC but not in oligometastatic disease; hypothesis, no direct supporting data.

**Table 5:** Registered trials of local therapy in oligometastatic and oligoprogressive NSCLC and the role of circulating tumor DNA in each.

Trial (Registry Number)	Population	Comparison	Primary Endpoint	Role of ctDNA
SARON (NCT02417662)	Synchronous oligometastatic NSCLC, 1 to 5 lesions in up to 3 organs, no actionable alteration	Systemic therapy with or without radiotherapy	Overall survival	None registered
ANDROMEDA (NCT06141070)	Stage IV NSCLC, 5 or fewer metastases, no driver eligible for targeted therapy	Standard systemic therapy with or without radiotherapy	Overall survival	None registered
TARGET-02 (NCT05278052)	Driver-negative NSCLC, 1 to 5 sites, no progression after 4 to 6 cycles of systemic therapy	Maintenance therapy with or without local consolidative radiotherapy	Overall survival	None registered
OliGRAIL (NCT06840782)	Synchronous oligometastatic NSCLC, 5 or fewer metastases in 3 or fewer organs, first-line immunotherapy-based treatment	Standard of care with or without radical local treatment	Overall survival	Secondary endpoint: ctDNA clearance at randomization, week 6 and month 4
LONESTAR (NCT03391869)	Stage IV NSCLC without progression after nivolumab and ipilimumab; oligometastatic subgroup with up to 3 metastases	Continued immunotherapy with or without local consolidation therapy	Overall survival	Secondary objective: baseline cell-free DNA profiles and clinical benefit and toxicity
NRG-LU002 (NCT03137771)	Metastatic NSCLC with 3 or fewer metastatic sites, without progression after first-line systemic therapy	Maintenance systemic therapy with or without local consolidative therapy	Progression-free survival (phase II); overall survival (phase III)	Secondary objective: correlation between ctDNA and clinical outcome
TARGET-01 (NCT05277844)	EGFR-, ALK- or ROS1-driven NSCLC, 1 to 5 sites, no progression on a tyrosine kinase inhibitor	Tyrosine kinase inhibitor with or without local consolidative radiotherapy	Progression-free survival	None registered
NorthStar (NCT03410043)	EGFR-mutant stage IIIB or IV NSCLC without progression after osimertinib induction; oligometastatic subgroup with up to 3 metastases	Osimertinib with or without local consolidative therapy	Progression-free survival	Exploratory objective: baseline cell-free DNA profiles and clinical benefit
Lazertinib trial (NCT05167851)	Treatment-naive synchronous oligometastatic EGFR-mutant NSCLC, 5 or fewer lesions	Lazertinib with or without early locally ablative radiotherapy	Progression-free survival	Exploratory secondary endpoint: resistance mechanisms in cell-free DNA at progression
HALT (NCT03256981)	Oligoprogression on a tyrosine kinase inhibitor, 5 or fewer extracranial sites	Stereotactic radiotherapy vs. none, inhibitor continued in both arms	Progression-free survival	Secondary endpoint: resistant subclones in ctDNA
NCT05429320 (non-randomized, completed)	Stage IV NSCLC, 10 or fewer lesions, first-line chemotherapy, immunotherapy or both	Local ablative therapy vs. continued systemic therapy, for a non-responding variant allele fraction without radiographic progression	Change in variant allele fraction; progression-free survival	Integral: defines eligibility (mean variant allele fraction falling by less than 50%) and the primary endpoint
NCT07410611 (single arm, not yet recruiting)	Treatment-naive EGFR-mutant oligometastatic NSCLC, 5 or fewer lesions in 3 or fewer organs	Befotertinib combined with radiotherapy	Progression-free survival	Stated to guide radiotherapy by dynamic ctDNA monitoring; rule not specified in the registry record
CURB-TKI (NCT06813664; single arm, not yet recruiting)	Oncogene-driven NSCLC on first-line tyrosine kinase inhibitor with up to 5 extracranial progressing sites	Stereotactic radiotherapy to progressing sites, followed by ctDNA analysis	Progression-free survival	Integral to the choice of subsequent targeted therapy, not to the decision for local therapy

Trials restricted to NSCLC with limited metastatic, oligometastatic, oligopersistent or oligoprogressive disease. Registry records on ClinicalTrials.gov accessed on 8 October 2026; trials identified from a recent review [33] and a registry search. None of the randomized trials uses ctDNA to select, stratify or allocate patients.

## Data Availability

No new data were created or analyzed in this study. Data sharing is not applicable to this article.
